# Supplementary material for: Patterns of Intron Gain and Loss in Fungi
Source: PLoS Biol. 2004 Nov 30;2(12):e422. doi: 10.1371/journal.pbio.0020422 (PMC532390; doi:10.1371/journal.pbio.0020422)
Supplement: Table S1 — Also available at http://genes.mit.edu/NielsenEtAl/. (4.3 MB ZIP). [file pbio.0020422.st001.zip › NielsenEtAl/html/785.html]

AN5901.1.NCU06322.1.MG01917.1.FG05247.1


```
 CLUSTAL W (1.82) Multiple Sequence Alignments - Introns Inserted


Sequence 1: NCU06322.1	273 aa
Sequence 2: MG01917.1	247 aa
Sequence 3: FG05247.1	250 aa
Sequence 4: AN5901.1	253 aa
Alignment Length: 289 aa
Number Identitical Residues: 91 aa
Alignment Score (without introns) 5222


MG01917.1 	MALPLPPGLTPSEIAFLCENELVTIVPRQRLESIPLLQ~GPTPTLRPPHRATLPLWLALL
NCU06322.1	MALPLPPGLTQNEVAFLAEMEMVTVVPRQRLDSIDLLG0GKTPQLRPPHRAQLPLWLALL
FG05247.1 	MALPLPSGLTPSEVAFLCEMELVTVVPRQRLESIELLT0GTTPALRPPHRSNLPLWLAIL
AN5901.1  	-------------------MELVTIVPRQRLEGLELLG0GPVAPLIPPRRTNVPLWLALL
          	                    *:**:******:.: **  * .. * **:*: :*****:*

MG01917.1 	LKKQRRANIIAPPWMHPASLQQIIQHETHTDP--VAFSPPPPPPS----RADGR--GGAR
NCU06322.1	LKKQRRANIVPPAWMHPASLAEIIHRETKEDP--EAFSPPPPPPS----RALYSQPGTAR
FG05247.1 	LKKQRRANIVPPPWLHPDSLRDIVHQETMVDR--KGWAPPPPPPA----RADSR--GNAR
AN5901.1  	LKRQRRANILPPPWLHPESLSLILDIETRDQAYQHAFSPPPPLPGQPSLRERSYKKSTAR
          	**:******:.*.*:** **  *:. **  :  . .::**** *...: *    . . **

MG01917.1 	RAN---------------IVGSTILSPPFLPSCTA--------ESPAGYLPYHWLEMAEI
NCU06322.1	RLNPSYLDDFTQTQQDSQQDPNSILSPPFLPSNVA--------ESPAGYLPYHWLEVAEA
FG05247.1 	NPF---------------MDDETVLSPPFLPSCTS--------DAPAGALPYHWFEVAEM
AN5901.1  	PRFTP-------------EGTKYYATPPFLPQNVAQDQAFDAFESEPPTLPFHWLEVGTM
          	   ..                .   :*****. .:...: .: :: .  **:**:*:.  

MG01917.1 	LLAHAPDDMPASAGEVRGLIRDLVEVRAAKMRASSADVEGFG--GGVMSLRGVGGAELAE
NCU06322.1	LLTHAGDDMPAPAGEVRSLLRDLVEVRAAKMRSSTSALEGFG--DAYLTLRGVGAMELAE
FG05247.1 	LLAHASDDISSSS-EVRSLLRDLQEVRAAKMRSSTAQLEGGV--DGVMSLRGVGAMELAE
AN5901.1  	LLDAAADDLVDPD-QTRRLLKELREVRSAKIRSGVEVLDDAAGPGGGVALTGVGAMEIGE
          	**  * **:  .  :.* *:::* ***:**:*:.   ::.  .... ::* ***. *:.*

MG01917.1 	NRGFVLGVVDGVR~KLGASTEATRREEEEAGR---------EDESDEDMGI
NCU06322.1	NRAFLAGLVDGVR~KIGASAEATRREEEEEARRGGDYGGDGDEDSDEDMGL
FG05247.1 	SRGFVIGVVEGVR~KLGASTETTRREEEEEGG-----GQESDEQSDEDMGL
AN5901.1  	GRGFISGVVDGLR2RIGASKEQARREQLADATN---REYDDMQDYNDEMEF
          	.*.*: *:*:*:* ::*** * :***:   .        .. :: :::* :
```
